# Contact Bioassays with Phenoxybenzyl and Tetrafluorobenzyl Pyrethroids against Target-Site and Metabolic Resistant Mosquitoes

**DOI:** 10.1371/journal.pone.0149738

**Published:** 2016-03-01

**Authors:** Sebastian Horstmann, Rainer Sonneck

**Affiliations:** Bayer CropScience AG, Monheim am Rhein, Germany; Institut Pasteur, FRANCE

## Abstract

**Background:**

Mosquito strains that exhibit increased tolerance to the chemical class of compounds with a sodium channel modulator mode of action (pyrethroids and pyrethrins) are typically described as “pyrethroid resistant”. Resistance to pyrethroids is an increasingly important challenge in the control of mosquito-borne diseases, such as malaria or dengue, because one of the main interventions (the distribution of large numbers of long-lasting insecticide-treated bed nets) currently relies entirely on long-lasting pyrethroids. Increasing tolerance of target insects against this class of insecticides lowers their impact in vector control. The current study suggests that the level of metabolic resistance depends on the structure of the molecule and that structurally different compounds may still be effective because detoxifying enzymes are unable to bind to these uncommon structures.

**Methods:**

Treated surface contact bioassays were performed on susceptible *Aedes aegypti*, East African *knockdown resistance* (*kdr) Anopheles gambiae* (strain RSP-H) and metabolically resistant *Anopheles funestus* (strain FUMOZ-R) with different pyrethroids, such as cypermethrin, ß-cyfluthrin, deltamethrin, permethrin and transfluthrin (alone and in combination with the synergist piperonyl butoxide). The nonfluorinated form of transfluthrin was also assessed as a single agent and in combination with piperonyl butoxide.

**Results:**

Although the dosages for pyrethroids containing a phenoxybenzyl moiety have exhibited differences in terms of effectiveness among the three tested mosquito species, the structurally different transfluthrin with a polyfluorobenzyl moiety remained active in mosquitoes with upregulated P450 levels. In trials with transfluthrin mixed with piperonyl butoxide, the added synergist exhibited no efficacy-enhancing effect.

**Conclusion:**

The results of this study suggest that transfluthrin has the potential to control P450-mediated metabolically resistant mosquitoes because the structural formula of transfluthrin differs from that of the tested pyrethroids, which are used in vector control. The P450-detoxifying enzymes of the *Anopheles funestus* FUMOZ-R mosquitoes seem to bind preferably at the phenoxybenzyl moiety and appear to be unable to degrade transfluthrin with its tetrafluorobenzyl moiety. Inhibition of the class of monooxygenases by piperonyl butoxide revealed no increase of efficacy of the pure transfluthrin compound, which also indicates that the P450 enzymes potentially do not impact the efficacy of transfluthrin.

## Background

Pyrethroids are a class of insecticides widely used in agriculture, household insecticides, professional pest control and vector control. In vector control applications, such as indoor residual sprays (IRS) or space sprays, pyrethroids play a major role. For long-lasting insecticidal nets (LLIN), pyrethroids are the only class of insecticides used [1, 2]. Most pyrethroids for IRS and LLIN, like cypermethrin or deltamethrin belong to the type II pyrethroids characterized by an α-cyano sidechain in the molecular structure. Additionally phenoxybenzyl groups are a characteristic element of these pyrethroids at the alcohol moiety. Permethrin is a type I pyrethroid, hence it does not contain an α-cyano- but a phenoxybenzyl group and is used for LLIN. The intensive use of pyrethroids and the consequential selection pressure on mosquito populations has led to the establishment of several resistance mechanisms, including physiological alterations, such as a thicker cuticle, target-site mutations at the corresponding receptor and increased detoxifying metabolism via specialized enzymes. Pyrethroids act at voltage-gated sodium channels and inhibit the closure of the pore after activation [3]. With target site mutations, the receptor to which the compound binds is morphologically altered by the exchange of one or more amino acids. This alteration reduces the binding speed and frequency of the respective compound. The most popular target-site mutation that affects pyrethroids is the so-called *knockdown resistance* (*kdr*) mutation, which occurs due to an amino acid exchange, especially at polypeptide sequence position 1014. Depending on the exchanged amino acid, the *kdr* is referred to as West or East African *kdr* due to its historical geographical distribution. The East African *kdr* carries a serine and the West *kdr* contains a phenylalanine instead of the wild-type amino acid leucine [4,5]. In *Aedes aegypti*, a special type of *kdr* is described, which is an exchange of a valine to isoleucine at position 1016 and a phenylalanine to cysteine substitution at position 1534 [6]. *Kdr* is also observed in several other insects that are targets of the public health sector, such as bed bugs [7], house flies and cockroaches [8]. The presence of *kdr* confers resistance with different resistance ratios, depending on the species in which the sodium channel is tested. For many insects, this ratio is between 5 and 20 for the L1014F mutation [9]. In a molecular model comparing this mutated form of the sodium channel with the wild type, the channel with the target-site resistance has been shown to be 10-fold less sensitive when cismethrin was applied and 17-fold less sensitive when deltamethrin was applied [10]. In this example a difference in sensitivity between type I and type II pyrethroids is measurable. This finding was supported by tests on genetically modified cockroach sodium channels. Here, depending on the type of target-site mutation a strong tolerance to type I pyrethroids was observed, whereas type II pyrethroids did not show differences to the wild-type channel reaction [11].

Another mechanism responsible for resistance in insects is the metabolic detoxification by specialized enzymes. These proteins are present in living organisms anyway and become a major resistance tool if the corresponding gene coding for such enzyme is duplicated and/or transcription amplification occurs [12]. Resistance takes place if increased amounts of these enzymes bind to the target molecule and trigger decomposition reactions. One major class of detoxifying enzymes are the P450 monooxygenases, which mainly confer resistance to pyrethroids and carbamates but also to organochlorines and organophosphates. Numerous different monooxygenases are involved in the detoxifying process depending on the insect species. In the resistant *Anopheles funestus* strain FUMOZ-R [13], two copies of the genes CYP6P4 and CYP6P9 are overexpressed 51- and 25-fold compared with the susceptible strain *Anopheles funestus* Angola (FANG) [14]. The corresponding enzymes play a role in pyrethroid detoxification. Stevenson *et al*. [15] identified 4-hydroxydeltamethrin as intermediate in the detoxification process of deltamethrin by the monooxygenase CYP6M2. This way of metabolizing deltamethrin started at the phenoxybenzyl moiety of the pyrethroid molecule. If the key-lock principle or induced-fit model of CYP6M2 depends on that molecule part, it is suspected that structural different pyrethroids may better withstand metabolism and remain active. 4-hydroxydeltamethrin was also detected as first degradation product in *Tribolium castaneum* [16]. For a strain of a cotton bollworm *Helicoverpa armigera* with upregulated P450 monooxygenases, Tan and McCaffery showed that structures with polyfluorobenzyl alcohols, lowered the resistance level [17]. The involved enzymes degrade the infiltrated insecticide and reduce or avoid its effect because only small amounts of the enzyme, if any at all, reach the target receptor. *Anopheles funestus* FUMOZ-R also exhibits morphological resistance due to cuticle thickness, which is increased in the described strain [18]. Lower uptake rates of insecticide might increase the efficacy of metabolic detoxification. This hypothesis is supported by findings of Fang *et al*. 2015, who found 14 cuticle proteins upregulated in deltamethrin-resistant *Culex pipiens* mosquitoes [19]. In the present study, we tested the efficacy response of a susceptible *Aedes aegypti* laboratory strain, an East African *kdr* resistant strain of *Anopheles gambiae* and the P450 resistant *Anopheles funestus* FUMOZ-R strain to three α-cyano pyrethroids with phenoxybenzyl moieties, deltamethrin, β-cyfluthrin and cypermethrin and the phenoxybenzyl pyrethroid permethrin that has no α-cyano group. Transfluthrin was selected as a pyrethroid with neither an α-cyano group nor a phenoxybenzyl moiety but with a polyfluorinated benzyl ring. By comparing the polyfluorinated transfluthin with the same chemical structure without fluorination, we generated data about the impact of fluorines on the degradation. The evidence of enhanced oxidative degradation was evaluated by testing mixtures of the pyrethroids and the non-fluorinated transfluthrin with the synergist piperonylbutoxide (PBO).

## Materials & Methods

### Materials

The technical grade pyrethroids cypermethrin (CAS No. 52315-07-8), permethrin (CAS No. 52645-53-1), ß-cyfluthrin (CAS No. 68359-37-5) and the synergist PBO (CAS No. 51-03-6) were purchased from Sigma-Aldrich® Chemical Company. Technical grade deltamethrin (CAS No. 52918-63-5) and transfluthrin (CAS No. 118712-89-3) were delivered by Bayer CropScience (BCS). The nonfluorinated transfluthrin molecule was synthesized by the Chemical Research department of Bayer CropScience.

### Insects

The mosquitoes *Anopheles gambiae* strain RSP-H (1), *Aedes aegypti* (2) and *Anopheles funestus* strain FUMOZ-R (3) were provided by Bayer CropScience insectary in Monheim, from permanent laboratory colonies.

1. *Anopheles gambiae* strain RSP-H is an East African *kdr* [L1014S] mosquito with upregulated amounts of a DDT-detoxifying glutathione-s-transferase (target-site and metabolic resistance). The strain is described in the reagent catalog of the Malaria Research and Reference Reagent Resource Center (www.MR4.org; MR4-number: MRA-334) as strain RSP (Resistant to permethrin). At MR4, the strain is challenged with permethrin every third generation. The strain was introduced into the authors’ rearing laboratories in 2006 without further challenging treatments. Since 2009, this strain has been reared as a 100% *kdr* strain, indicating that newly eclosed *Anopheles*-adults are sorted out of the hatching population and one leg from each adult is analyzed genetically for the presence of the *kdr* mutation. Afterwards, the *kdr-*positive mosquitoes are pooled for mating and further rearing. The frequency of the *kdr* mutation in the population is therefore 100%. According to the MR4 website, the RSP strain contains elevated P450 enzyme levels, which are therefore also elevated in the RSP-H strain, even if this strain was not reared under permethrin selection pressure.
2. *Aedes aegypti* is a susceptible strain that exhibits neither a measurable upregulation of detoxifying enzymes, such as monooxygenases, glutathione-s-transferases or esterases, nor any described target-site mutations (unpublished Bayer CropScience data). The use of an *Aedes* species as a susceptible strain for comparison in this study is based on limited rearing capacities that do not allow the rearing of several *Anopheles* species. A susceptible *Anopheles* strain was therefore not available.
3. *Anopheles funestus* strain FUMOZ-R overexpresses P450 monooxygenases, which are involved in the detoxification of pyrethroids. This strain lacks any known target-site mutation [12]. The strain was introduced in 2011 into Bayer CropScience rearing department coming from the University of the Witwatersrand, South Africa.

### Methods

#### Bioassay

The mosquitoes with an imago age of 2 to 3 days were anesthetized with carbon dioxide. Ten anesthetized mosquitoes of mixed sex were sorted into a perforated Petri dish, which was subsequently closed by placing a white paper card on top and flipping it over to serve as a small, mobile unit containing 10 mosquitoes. The mosquitoes remained in this mobile unit for at least one hour to minimize the impact of the anaesthetization on the physiology of the insects. The glazed-tile bioassay is a standard procedure for compound screening in our laboratories and therefore is well established. Further references concerning this testing method can be found in the literature [20, 21, 22, 23, 24, 25].

**Fig 1:**
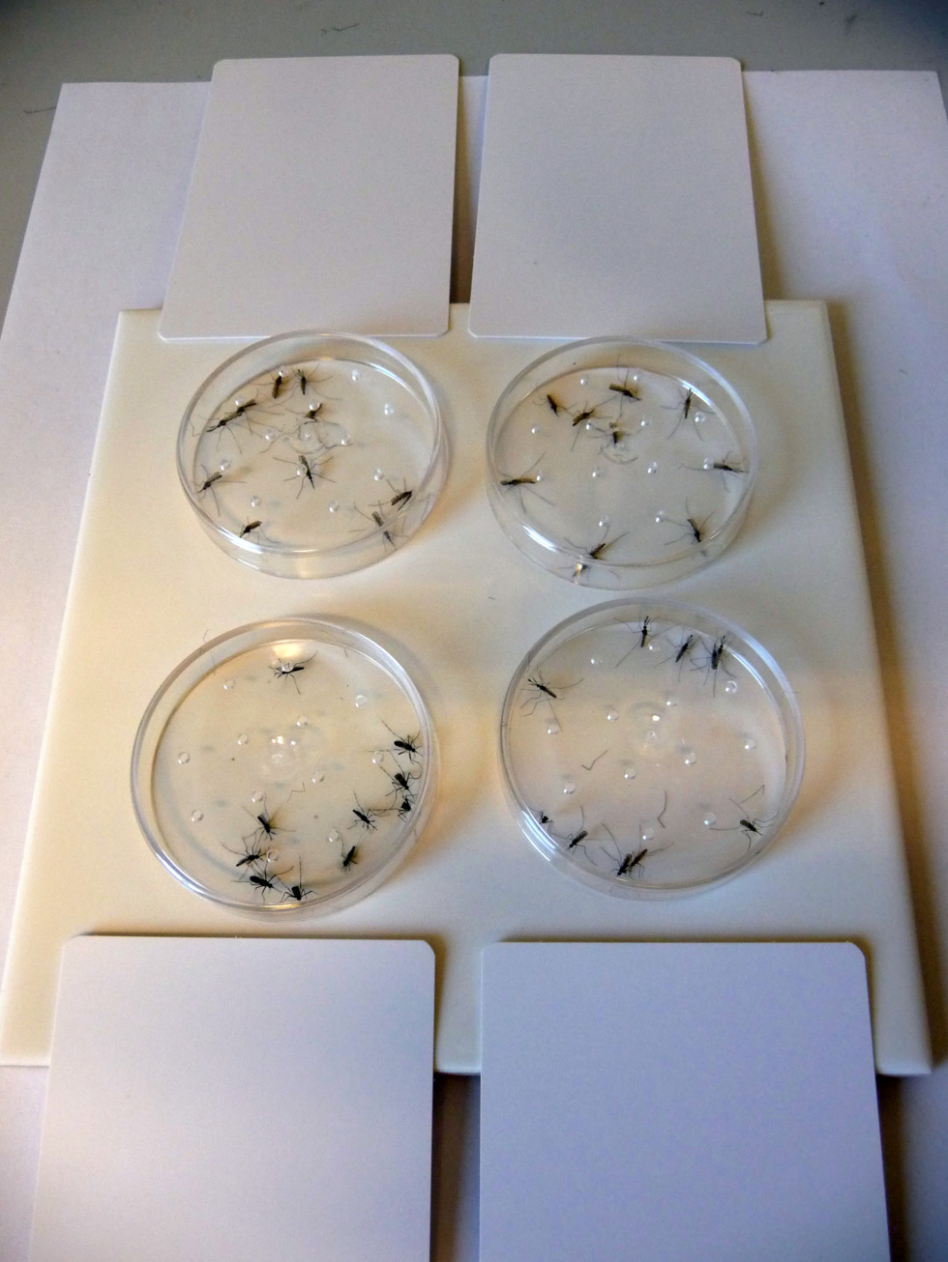
The arrangement of the mosquito containing Petri dishes on a glazed tile with an active ingredient-treated surface.

**Fig 2:**
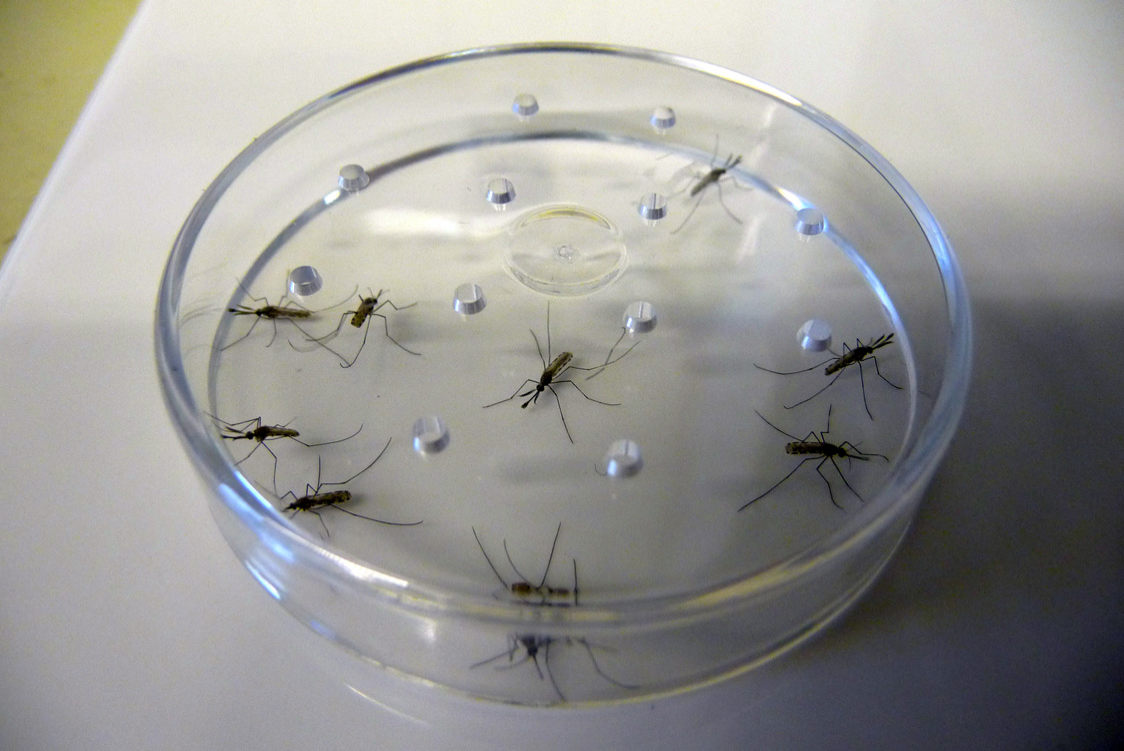
The close-up view reveals the contact of the test insect with the treated surface of the tile.

The technical grade active ingredients were dissolved in acetone and equally distributed with a pipette onto the surface of a glazed tile (15 cm x 15 cm). The active ingredient acetone solution was prepared at a ratio of 2 mg/ml, which after application of 1250 μl onto the tile (0.0225 m^2^) provided a concentration of 100 mg ai/m^2^. The 2 mg/ml stock solution was used to apply 100 mg ai/m^2^ to the tile and for the preparation of the other concentrations (1:5 ratios; 20, 4, 0.8, and 0.16 mg ai/m^2^). The tile treatments as well as handling of the other compounds were performed in a fume hood. Acetone (1250 μl) was applied to a tile to serve as a negative control. After application, the acetone was allowed to evaporate for at least 30 min. Then, the test insects were exposed to the tiles for 30 min. Contact with the treated surface of the tile was ensured by placing the mobile unit on the tile and removing the white paper card. The exposure time was stopped by re-inserting the paper card. Four Petri dishes were placed on one tile (Fig 1). Ten *Aedes aegypti* mosquitoes, ten *Anopheles funestus* FUMOZ-R mosquitoes (Fig 2) and twenty *Anopheles gambiae* strain RSP-H mosquitoes were tested for each approach; two replicates of this test were performed. Therefore, the average data for *Aedes aegypti* and *Anopheles funestus* are based on two replicates, whereas the values for *Anopheles gambiae* are based on four replicates. The white paper card was removed from below the Petri dishes after the exposure procedure.

The contact activity was observed during the first 30 min of exposure time. After the exposure, the mosquitoes were transferred to an observation table, where they remained for 24 hours. The number of mosquitoes that were knocked down and/or died was recorded after one hour.

In trials with PBO, a stock solution of acetone and 1600 ppm PBO was prepared. This stock solution was used for the 2 mg/ml mixture with the active ingredient. The 1600 ppm also remained constant for dilutions of the active ingredients. Thus, in dilutions of the active ingredient, PBO was not diluted in the same manner.

#### ED_50_ and efficacy ratios

For a side-by-side comparison of the pyrethroid´s activity with and without PBO on the three mosquito strains we established the ED_50_ in mg/m^2^ (effective dose to knock down or kill 50%) and the 95% confidence intervals for each substance and strain one hour after contact to the treated surface. The term “effective dose” was used to comprise knockdown and dead mosquitoes. The relative efficacy of the tested pyrethroids to transfluthrin, all with and without PBO, is expressed in form of efficacy ratios. To obtain the efficacy ratio, the calculated ED_50_ of each tested pyrethroid is divided by the transfluthrin ED_50_ concentration. Transfluthrin represents an efficacy ratio of 1. Factors higher than 1 indicate an increased tolerance of the tested mosquito strain to the tested pyrethroid compared to transfluthrin.

All statistical analyses, including control efficacy corrections according to Abbot´s formula and the Probit analysis, were calculated in the software program *ToxRat® Standard*, *version 2*.*10*, ToxRat Solutions GmbH, Naheweg 15, D-52477 Alsdorf, Germany, which is a good laboratory practice (GLP)-certified statistical program [26].

## Results

Table 1 shows the effective dose 50% (ED_50_) in mg/m^2^ and the confidence intervals CI [lower 95%—upper 95%] of all tested active ingredients with and without PBO on three mosquito strains. The *p*-values of a one-sided Student´s t-test comparing the measured arithmetic means of selected compound data for significant differences are given in Table 2.

**Table 1:** ED_50_ values with confidence intervals

Substance	*Aedes aegypti*		*Anopheles gambiae RSP-H*		*Anopheles funestus FUMOZ_R*	
	ED_50_ (mg/m^2^)	CI	ED_50_ (mg/m^2^)	CI	ED_50_ (mg/m^2^)	CI
*deltamethrin*	0.0015	[n.d.]	0.0318	[0.0223–0.0451]	14.835	[0.793–2.8119]
*deltamethrin + PBO*	0.0032	[0.0022–0.0044]	0.0045	[0.0034–0.0059]	0.0031	[0.002–0.0047]
*ß-cyfluthrin*	0.0042	[0.0027–0.0064]	0.0112	[0.0085–0.0149]	0.8616	[0.194–3.8645]
*ß-cyfluthrin + PBO*	0.0046	[n.d.]	0.0033	[0.0022–0.0047]	0.0046	[0.0031–0.0070]
*cypermethrin*	0.006	[n.d]	0.0164	[0.0102–0.0255]	17.906	[0.9049–3.528]
*cypermethrin + PBO*	0.011	[0.0077–0.0162]	0.009	[0.0065–0.0125]	0.0047	[0.0028–0.0077]
*permethrin*	0.0691	[0.0439–0.1088]	0.3903	[0.1827–0.8337]	0.8258	[n.d.]
*permethrin + PBO*	0.0346	[n.d.]	0.2662	[0.1138–0.622]	0.1781	[0.1178–0.2693]
*transfluthrin*	0.0021	[0.0007–0.0039]	0.004	[0.0029–0.0055]	0.0019	[0.0007–0.0034]
*transfluthrin + PBO*	0.0049	[0.0031–0.0075]	0.0104	[0.0077–0.0141]	0.0046	[n.d.]
*transfluthrin without fluorination*	0.578	[0.446–1.201]	16.045	[1.199–2.1562]	0.7322	[0.4464–1.2011]
*transfluthrin without fluorination + PBO*	0.0411	[0.027–0.062]	0.1859	[n.d.]	0.0552	[0.0386–0.0812]

Table 1: Probit analysis data showing the effective dose 50% (ED_50_) in mg/m^2^ and the confidence intervals CI [lower 95%—upper 95%].

**Table 2:** *p*-values of a one-sided Student´s t-test comparing the arithmetic means of different samples.

Arithmetic means sample 1 / sample 2	*p*-values (α = 0.05)		
	*A*. *aegypti*	*A*. *gambiae*	*A*. *funestus*
*deltametdrin / deltametdrin + PBO*	0.385	0.208	0.010*
*deltamethrin / transfluthrin*	0.397	0.190	0.005*
*deltamethrin + PBO / transfluthrin*	0.465	0.484	0.425
*ß-cyfluthrin / ß-cyfluthrin + PBO*	0.457	0.307	0.028*
*ß-cyfluthrin / transfluthrin*	0.357	0.331	0.012*
*ß-cyfluthrin + PBO / transfluthrin*	0.450	0.474	0.367
*cypermethrin / cypermethrin + PBO*	0.389	0.412	0.015*
*cypermethrin / transfluthrin*	0.357	0.271	0.003*
*cypermethrin + PBO / transfluthrin*	0.257	0.361	0.326
*permethrin / permethrin + PBO*	0.427	0.456	0.319
*permethrin / transfluthrin*	0.107	0.047*	0.014*
*permethrin + PBO / transfluthrin*	0.142	0.053	0.051
*transfluthrin / transfluthrin + PBO*	0.365	0.331	0.416
*transfluthrin without fluorination / transfluthrin without fluorination + PBO*	0.211	0.274	0.202
*transfluthrin without fluorination / transfluthrin*	0.027*	0.023*	0.017*
*transfluthrin without fluorination + PBO / transfluthrin*	0.131	0.079	0.105

Table 2: Calculated *p*-values of a one-sided Student´s t-test (H_0_ = the measured values are randomly different; H_1_ = the measured values are significantly different; *p*<0.05 means H_1_ will be accepted–marked with *).

**Fig 3:**
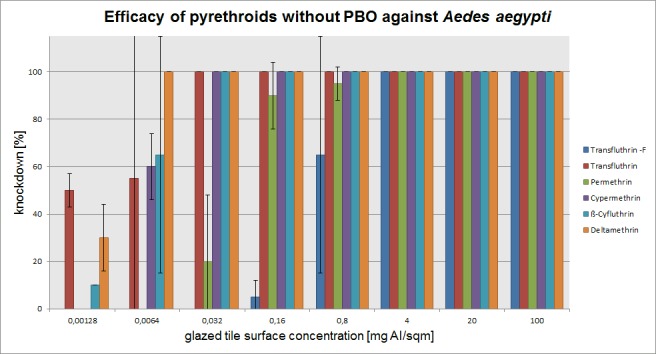
Arithmetic means and standard deviations of the 1 h results of a glazed tile contact bioassay using technical grade pyrethroids without PBO against insecticide susceptible *Aedes aegypti*.

**Fig 4:**
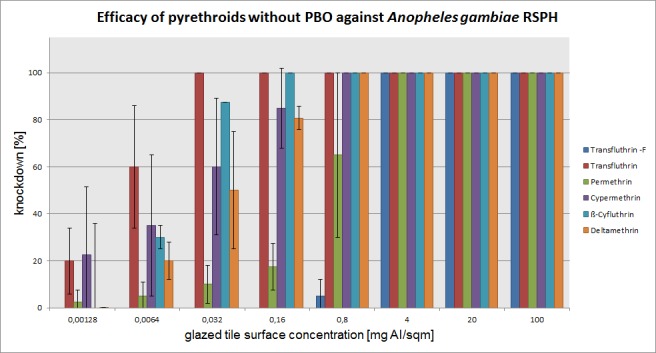
Arithmetic means and standard deviations of the 1 h results of a glazed tile contact bioassay using technical grade pyrethroids without PBO against the *kdr-*containing *Anopheles gambiae* RSP-H strain.

**Fig 5:**
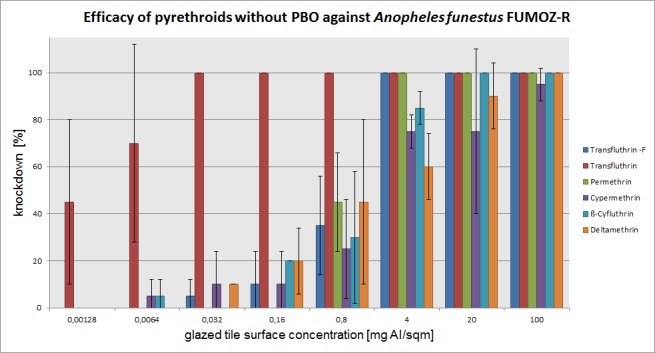
Arithmetic means and standard deviations of the 1 h results of a glazed tile contact bioassay using technical grade pyrethroids without PBO against the metabolic resistant *Anopheles funestus FUMOZ-R* strain.

**Fig 6:**
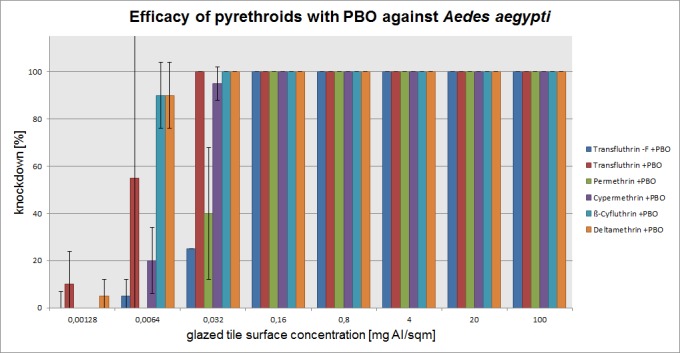
Arithmetic means and standard deviations of the 1 h results of a glazed tile contact bioassay using technical grade pyrethroids with PBO (1600ppm) against insecticide susceptible *Aedes aegypti*.

**Fig 7:**
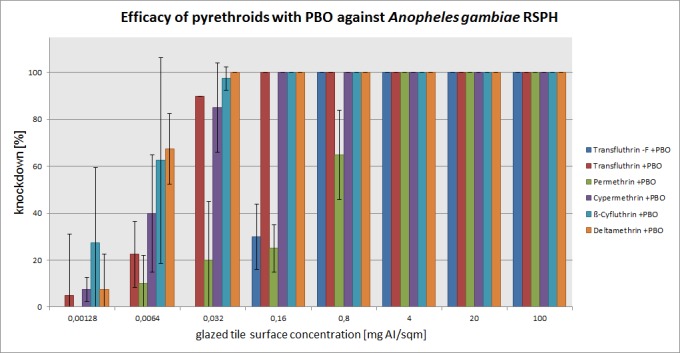
Arithmetic means and standard deviations of the 1 h results of a glazed tile contact bioassay using technical grade pyrethroids with PBO (1600 ppm) against the *kdr-*containing *Anopheles gambiae* RSP-H strain.

**Fig 8:**
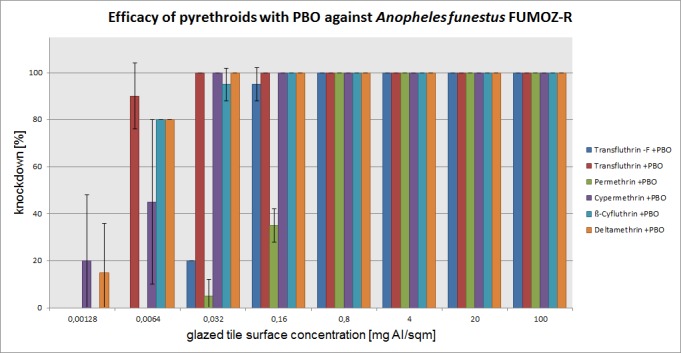
Arithmetic means and standard deviations of the 1 h results of a glazed tile contact bioassay using technical grade pyrethroids with PBO (1600 ppm) against the metabolic resistant *Anopheles funestus FUMOZ-R* strain.

The mean percentage of knockdown after one hour, recorded for each active ingredient concentration (without PBO) on three mosquito species is presented in Figs 3–5. The test results with PBO are shown in Figs 6–8. The overall efficacy of the tested active ingredients among the mosquito species revealed differences in the calculated median effective dose (ED_50_) concentrations and with the addition of PBO, with the exception of transfluthrin.

Table 3 gives the calculated efficacy ratios of the pyrethroids and PBO mixtures for each tested mosquito strain. Transfluthrin ED_50_ value was used as divisor of ED_50_ of the respective pyrethroid.

**Table 3:** Calculated efficacy ratios.

Substance	Efficacy ratio (Values divided by the transfluthrin ED_50_)		
	*A*. *aegypti*	*A*. *gambiae*	*A*. *funestus*
*deltametdrin*	1	8	781
*deltamethrin + PBO*	2	1	2
*ß-cyfluthrin*	2	3	454
*ß-cyfluthrin + PBO*	2	1	2
*cypermethrin*	3	4	942
*cypermethrin + PBO*	5	2	2
*permethrin*	33	98	435
*permethrin + PBO*	17	67	94
*transfluthrin*	1	1	1
*transfluthrin + PBO*	2	3	2
*transfluthrin without fluorination*	275	401	385
*transfluthrin without fluorination + PBO*	20	46	29

Table 3: Calculated efficacy ratios for each tested mosquito strain. Transfluthrin ED_50_ value was used as divisor of ED_50_ of the respective pyrethroid.

### Aedes aegypti

The pyrethroids tested without PBO were effective against the susceptible *Aedes aegypti* mosquito down to the dose rate of 0.0032 mg/m^2^, with the exception of the nonfluorinated transfluthrin. The differences between the compound with PBO and the active ingredient alone are rather low. Deltamethrin was the only pyrethroid that provided 100% control of *Aedes aegypti* still at a concentration of 0.0064 mg/m^2^. For the other pyrethroids the dilution step to 0.0064 mg/m^2^ revealed a clear loss of activity. The efficacy at that dose rate did not exceed 65% knockdown. Adding PBO to 0.0064 mg/m^2^ ß-cyfluthrin resulted in an increase to 100% knockdown but a decrease in the efficacy of deltamethrin by 10% and cypermethrin by 40%. PBO appeared to have an effect on 0.032 mg/m^2^ permethrin but especially on the nonfluorinated transfluthrin molecule. Here, 0.16 mg/m^2^ is sufficient for 100% knockdown of the test insects, whereas 4 mg/m^2^ must be used in the absence of PBO. These findings were also reflected in the comparison of the efficacy ratios for *Aedes aegypti*. To obtain the efficacy ratio, the calculated ED_50_ of each tested pyrethroid were divided by the concentration of the transfluthrin ED_50_ concentration. By performing this calculation, an efficacy ratio of 1 represents transfluthrin itself as well as other pyrethroids with a similar effective dose against the same insect species. For example, the following equation was used to calculate the efficacy ratio as shown in a comparison of transfluthrin and the nonfluorinated variant of that molecule in *Aedes aegypti*: (nonfluorinated transfluthrin [0.578] transfluthrin [0.0021] = 0.578/0.0021 = 275). The dosage of the nonfluorinated transfluthrin that is necessary for 50% knockdown after 1 hour is increased 275-fold compared with transfluthrin. High efficacy ratios indicate therefore a high tolerance of this mosquito strain to a certain pyrethroid and if the ratio can be lowered by addition of PBO it indirectly shows the influence of P450 monooxygenases.

In the case of *Aedes aegypti*, the efficacy ratios are considerably increased for permethrin and the nonfluorinated version of transfluthrin with and without PBO.

### *Anopheles gambiae* RSP-H

Concerning *Anopheles gambiae* RSP-H only transfluthrin obtained 100% knockdown at 0.032 mg/m^2^. Deltamethrin, cypermethrin and ß-cyfluthrin knocked down 50%, 60% and 87.5% of *Anopheles gambiae* RSP-H. Here, the enhancing effects of PBO were observed with regard to deltamethrin, cypermethrin and ß-cyfluthrin with 100%, 85% and 97.5% efficacy. PBO exhibited synergistic effects on the nonfluorinated transfluthrin molecule at 0.8 mg/m^2^. Synergistic effects on permethrin remained low. *Anopheles gambiae* RSP-H resisted the treatment of permethrin and the nonfluorinated transfluthrin molecule, with and without PBO. Consequently the efficacy ratios of both substances were increased. While PBO only slightly reduced the ratio of permethrin from 98 to 67, the ratio of the nonfluorinated transfluthrin decreased from 401 to 46.

### *Anopheles funestus* FUMOZ-R

The greatest differences in tests with and without PBO were observed in *Anopheles funestus* FUMOZ-R. Deltamethrin, cypermethrin and ß-cyfluthrin containing an α-cyano sidechain and a phenoxybenzyl ring in the molecular structure started to lose efficacy below 95% at 20 mg/m^2^ and 4 mg/m^2^. As PBO was added to these pyrethroids, efficacy was fully recovered at a dosage of 0.032 mg/m^2^ to provide 100% knockdown. The dose rate of permethrin and the nonfluorinated transfluthrin was 0.8 mg/m^2^. PBO helped at that dose rate to increase efficacy to 100%. While PBO synergized the nonfluorinated transfluthrin at the dose rate of 0.16 mg/m^2^ to obtain 95% knockdown, the synergistic effect to permethrin was insufficient. In the case of transfluthrin the active ingredient concentrations for 100% knockdown with and without PBO were 0.032 mg/m^2^. A dose rate of 0.0064 mg/m^2^ resulted in 70% efficacy and was increased up to 90% by adding PBO. Efficacy below 50% was recorded at the lowest tested dose rate of 0.00128 mg/m^2^ with transfluthrin. The addition of PBO to that dose rate resulted in a complete loss of efficacy. The efficacy data on *Anopheles funestus* FUMOZ-R mosquitoes revealed increased efficacy ratios between the lowest (nonfluorinated transfluthrin) to the highest 942 (cypermethrin) for the straight pyrethroids. However, PBO showed a severe impact on the efficacy of the type II pyrethroids deltamethrin, cypermethrin and ß-Cyfluthrin. If PBO was added to these pyrethroids, the metabolic degradation was suppressed to an efficacy ratio of 2. The ratios of PBO to permethrin and the nonfluorinated transfluthrin were less dominant.

## Discussion

As expected, when applied to the susceptible *Aedes aegypti* strain, low concentrations of all pyrethroid molecules achieved 50% knockdown (Table 1). Comparing the ED_50_ values of the *Aedes* column, permethrin and nonfluorinated transfluthrin required relatively higher dosages (Table 1). The combination with PBO resulted in lower ED_50_ concentrations only for these two molecules, which may indicate an involvement of baseline P450. The same applies to the *Anopheles gambiae* RSP-H strain. Since, with the exception of transfluthrin, all ED_50_ values of the tested pyrethroids are reduced by adding PBO, it is rather likely that specialized P450 enzymes are upregulated in this mosquito strain on top of the target-site resistance (Table 1). This assumption is supported by the calculated efficacy ratios that exhibit higher values for the straight compounds. The addition of PBO lowered the efficacy ratios, in the case of deltamethrin and ß-cyfluthrin to the transfluthrin level. A notable influence of the target-site resistance could not be measured and a comparison between the tested mosquito strains would be imprecise as the insects belong to different genera.

Resistance factors conferred by the *kdr* mutation are described as uniform at approximate values of 14 [27] or 20 to 50 [28]. Higher resistance factors have been observed between susceptible and metabolically resistant mosquito strains, *e*.*g*., a 2500-fold increased resistance to permethrin compared with a susceptible strain in *Culex quinquefasciatus* [29].

However, given that the two mechanisms included in this trial can act independently, a combination of target-site and metabolic resistance would represent a major hurdle for effectiveness of an insecticide. To overcome strong resistance to pyrethroids, active ingredients with different modes of action or mixtures of active compounds with synergists, such as PBO, are used, and their use has been the main strategy to date. The *Anopheles funestus* strain FUMOZ-R is described as highly pyrethroid resistant [13, 30] and the measured ED_50_ values in this study confirmed this (Table 1).

High efficacy ratios were noted with all tested pyrethroids in the FUMOZ-R strain (Table 3). Clearly, these pyrethroids can be more easily detoxified than transfluthrin. The resistance can be overcome by using PBO, which again suggests the involvement of P450 enzymes. In this case the ratios for deltamethrin, ß-cyfluthrin and cypermethrin were brought down to the transfluthrin level (Table 3). These results were supported by the test for significance that revealed differences in comparison of the single pyrethroids with their synergized variant or with transfluthrin (Table 2). Differences in the comparison of the synergized pyrethroid with transfluthrin are caused by randomness except for the data of permethrin and nonfluorinated transfluthrin. These two active ingredients are of lower toxicity than transfluthrin itself.

**Fig 9:**
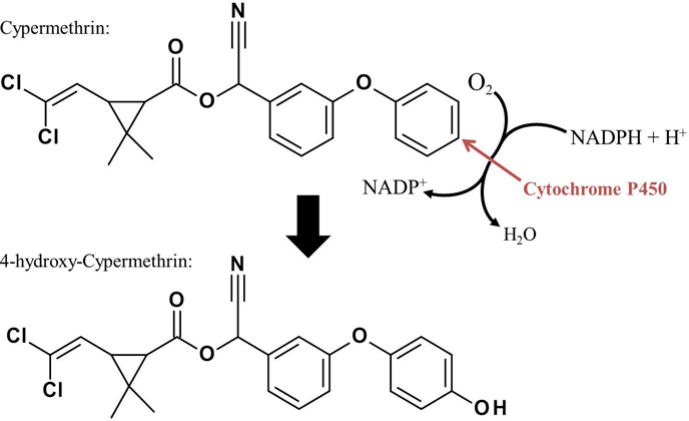
In pyrethroids with a phenoxybenzyl moiety, P450 enzymes catalyze the hydroxylation from, *e*.*g*., cypermethrin to 4-hydroxy-cypermethrin. The hydroxylation reaction occurs under usage of oxygen and the cofactor NADPH.

If PBO does not synergize transfluthrin, as our study indicates, the P450 monooxygenases potentially are not involved in the detoxification processes of this molecule (Tables 1 and 3). The addition of PBO slightly increased the efficacy ratio but this effect is not significant (Table 2). The reason for the inability of metabolic degradation of transfluthrin in *Anopheles funestus* FUMOZ-R could be the molecular structure, which is unlike other common pyrethroids: the alcoholic sidechain contains a tetrafluorobenzyl ring, whereas most common pyrethroids show a phenoxybenzyl ring. An intermediate in the detoxification process of deltamethrin is 4-hydroxydeltamethrin [15, 16] and a similar path of detoxification was confirmed for permethrin [31]. According to these findings, the metabolism of deltamethrin and permethrin starts at the phenoxybenzyl moiety of the molecule involving P450 enzymes. The monooxygenase CYP6D1 was found to be part of deltamethrin degradation in houseflies and it was further described that this detoxification occurred at a single site at the phenoxybenzyl moiety [32]. The same effect was observed with cypermethrin when 4-hydroxycypermethrin was identified as a metabolite by gas chromatography/mass spectrometry [32]. A schematic representation about this first step in an enzyme triggered decomposition cascade with the aim to detoxify and excrete cypermethrin is shown below (Fig 9).

In trials with *Culex quinquefasciatus* strains from Saudi Arabia with upregulated P450, high levels of resistance were described for pyrethroids with phenoxybenzyl moieties [33]. Interestingly, this type of detoxification is not restricted to one class of insecticides. The mechanism also works for the class of juvenile hormone mimics, where monooxygenases are involved in the degradation of pyriproxyfen in white flies [34] and microsomal extracts of houseflies [35]. The main metabolites here were 4- and 5-hydroxypyriproxyfen, suggesting once again a detoxification that starts at the phenoxybenzyl moiety of this compound.

**Fig 10:**
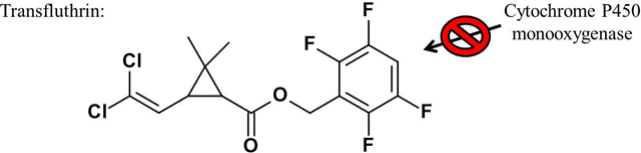
P450 enzymes are potentially unable to use transfluthrin with a polyfluorobenzyl ring as suitable substrate for the P450-mediated way of detoxification.

Data presented in this study show transfluthrin as highly effective pyrethroid against *Anopheles funestus* FUMOZ-R. The activity indicates the inability of monooxygenases to detoxify this structurally different pyrethroid that lacks a phenoxybenzyl moiety. The addition of PBO did not increase the activity, which supports the hypothesis that P450 enzymes are not involved in the detoxification processes of transfluthrin. To test whether this is based on the fluorination of the benzoyl ring of transfluthrin, a molecule with a similar structure but without fluorination was constructed. The efficacy of this nonfluorinated transfluthrin-like molecule was reduced in all tested mosquito strains, indicating that the P450 enzymes were now able to detoxify the molecule to a greater extent. The combination of PBO with nonfluorinated transfluthrin restored the efficacy and therefore lowered the ED_50_ values and efficacy ratios (Tables 1 and 3). A hypothetic hydroxylation scheme for the nonfluorinated transfluthrin can be found in S1 Fig. The tetrafluoro substituents on the benzyl ring of transfluthrin appear to act as a shield, protecting it from the degradation process (Fig 10). Transfluthrin exhibits a favorable toxicological profile in mammals, indicating effective detoxification or degradation mechanisms. Current findings [36] suggest that in mammals, the first metabolites are created by an esterase activity that cleaves transfluthrin into the corresponding acid and alcohol molecules (*e*.*g*., 2,3,5,6-tetrafluorobezyl alcohol and 3-(2,2-dichlorovinyl)-2,2-dimethylcyclopropane carboxylic acid (DCCA)). These molecules as well as further hydrolyzed metabolites are water soluble and can be rapidly eliminated via the urinary system [36]. If these esterases were identified in mosquitoes, they would probably also be able to degrade transfluthrin in the same manner as other common pyrethroids because the esterase cleavage site is present in almost all pyrethroids, except etofenprox. Another resistance mechanism in the tested *Anopheles funestus* FUMOZ-R strain has been described as a thicker cuticle on the legs and tarsi, which represents a greater barrier for the insecticide to enter the insect body [18]. One hypothesis is that the high volatility of transfluthrin allows the compound to enter the insect via the respiratory system where it could therefore bypass this resistance mechanism.

The results of these experiments illustrate that transfluthrin was unaffected by the pyrethroid detoxification enzymes from the class of monooxygenases found in *Anopheles funestus* FUMOZ-R and *Anopheles gambiae* RSP-H. This finding is possibly attributable to its different structural formula compared with the other pyrethroids tested (most of which are commonly used in vector control). Further investigation is needed to support this hypothesis and confirm the potential for transfluthrin to be effective against metabolically resistant mosquitoes in field trials. Given that several published studies have already suggested that P450-mediated detoxification is very common in insects (and notably, in *Anopheles* malaria vectors), transfluthrin could become an important resistance management tool to help overcome this type of metabolic resistance.

## Supporting Information

S1 Fig Hypothetical hydroxylation scheme for the nonfluorinated transfluthrin molecule. (TIF)

S1 Table Knockdown/mortality results of the glazed tile contact bioassay using technical grade type II pyrethroids (surface concentration shown in the respective row) with and without the addition of piperonyl butoxide (1600 ppm) against three different mosquito strains. (PDF)

S2 Table Knockdown/mortality results of the glazed tile contact bioassay using technical grade type I pyrethroids (surface concentration shown in the respective row) with and without the addition of piperonyl butoxide (1600 ppm) against three different mosquito strains. (PDF)
