# Supplementary figures and images for: Contact Bioassays with Phenoxybenzyl and Tetrafluorobenzyl Pyrethroids against Target-Site and Metabolic Resistant Mosquitoes
Source: PLoS One. 2016 Mar 1;11(3):e0149738. doi: 10.1371/journal.pone.0149738 (PMC4773128; doi:10.1371/journal.pone.0149738)

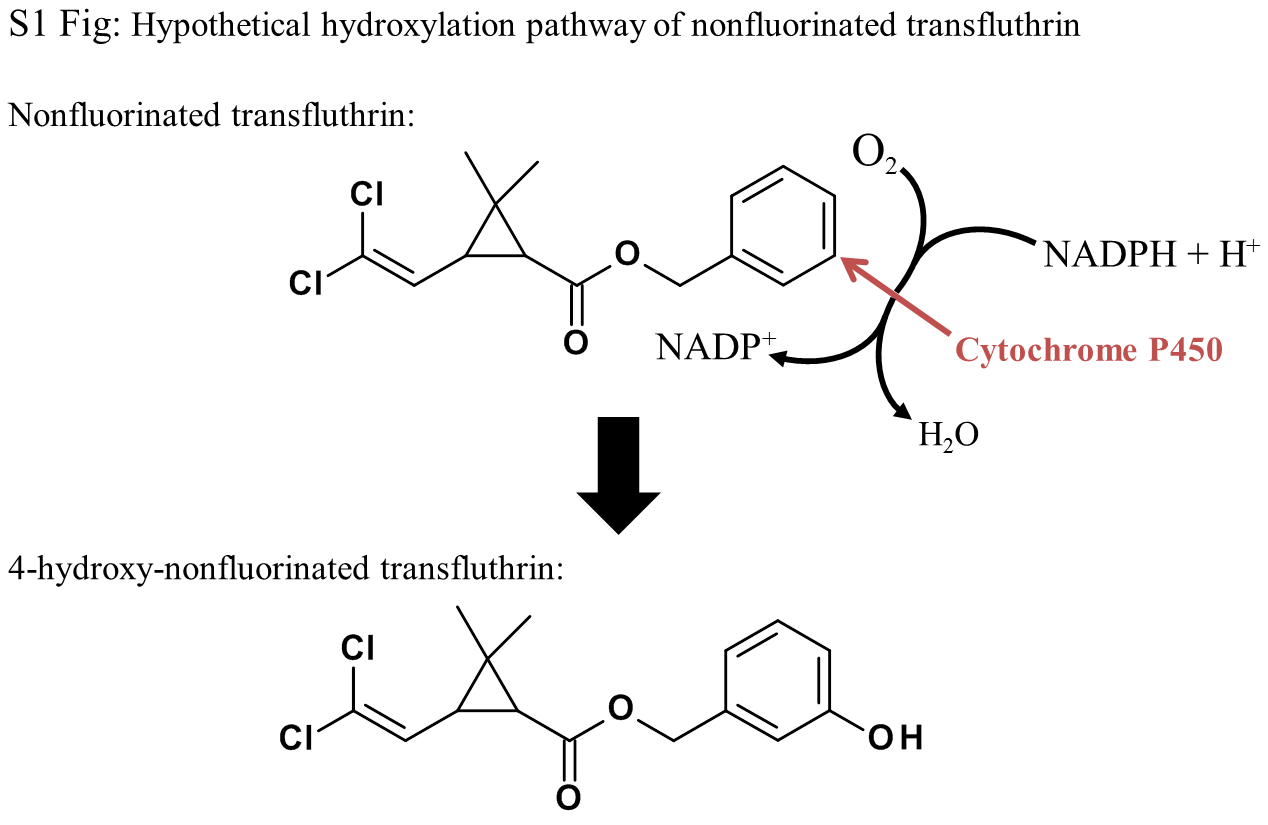

Supplement: S1 Fig — (TIF) [file pone.0149738.s001.tif]
