# Supplementary material for: Contact Bioassays with Phenoxybenzyl and Tetrafluorobenzyl Pyrethroids against Target-Site and Metabolic Resistant Mosquitoes
Source: PLoS One. 2016 Mar 1;11(3):e0149738. doi: 10.1371/journal.pone.0149738 (PMC4773128; doi:10.1371/journal.pone.0149738)
Supplement: S1 Table — (PDF) [file pone.0149738.s002.pdf]

S1 Table: *Knockdown* results of the glazed tile contact bioassay using technical grade type II pyrethroids (surface concentration shown in the respective row) with and without the addition of piperonyl butoxide (1600 ppm) against three different mosquito strains.

	concentration [mg/m²]	*deltamethrin* % knockdown 1 hour after contact	*deltamethrin* + PBO % knockdown 1 hour after contact	*ß-cyfluthrin* % knockdown 1 hour after contact	*ß-cyfluthrin* + PBO % knockdown 1 hour after contact	*cypermethrin* % knockdown 1 hour after contact	*cypermethrin* + PBO % knockdown 1 hour after contact
Aedes aegypti [Monheim]	200	n.t.	n.t.	n.t.	n.t.	100	100
	100	100	100	100	100	100	100
	20	100	100	100	100	100	100
	4	100	100	100	100	100	100
	0.8	100	100	100	100	100	100
	0.16	100	100	100	100	100	100
	0.032	100	100	100	100	100	95
	0.0064	100	90	65	90	60	20
	0.00128	30	5	10	0	0	0
An. gambiae [RSPH]	200	n.t.	n.t.	n.t.	n.t.	100	100
	100	100	100	100	100	100	100
	20	100	100	100	100	100	100
	4	100	100	100	100	100	100
	0.8	100	100	100	100	100	100
	0.16	80.75	100	100	100	85	100
	0.032	50	100	87.5	97.5	60	85
	0.0064	20	67.5	30	62.5	35	40
	0.00128	0.25	7.5	0	27.5	22.5	7.5
An. funestus [FUMOZ-R]	200	n.t.	n.t.	n.t.	n.t.	100	100
	100	100	100	100	100	95	100
	20	90	100	100	100	75	100
	4	60	100	85	100	75	100
	0.8	45	100	30	100	25	100
	0.16	20	100	20	100	10	100
	0.032	10	100	0	95	10	100
	0.0064	0	80	5	80	5	45
	0.00128	0	15	0	0	0	20
