# Supplementary material for: Contact Bioassays with Phenoxybenzyl and Tetrafluorobenzyl Pyrethroids against Target-Site and Metabolic Resistant Mosquitoes
Source: PLoS One. 2016 Mar 1;11(3):e0149738. doi: 10.1371/journal.pone.0149738 (PMC4773128; doi:10.1371/journal.pone.0149738)
Supplement: S2 Table — (PDF) [file pone.0149738.s003.pdf]

S2 Table: *Knockdown* results of the glazed tile contact bioassay using technical grade type I pyrethroids (surface concentration shown in the respective row) with and without the addition of piperonyl butoxide (1600 ppm) against three different mosquito strains.

	concentration [mg/m^2]	*permethrin* % knockdown 1 hour after contact	*permethrin* + PBO % knockdown 1 hour after contact	*transfluthrin* % knockdown 1 hour after contact	*transfluthrin* + PBO % knockdown 1 hour after contact	nonfluorinated *transfluthrin* % knockdown 1 hour after contact	nonfluorinated *transfluthrin* + PBO % knockdown 1 hour after contact
Aedes aegypti [Monheim]	200	n.t.	n.t.	n.t.	n.t.	n.t.	n.t.
	100	100	100	100	100	100	100
	20	100	100	100	100	100	100
	4	100	100	100	100	100	100
	0.8	95	100	100	100	65	100
	0.16	90	100	100	100	5	100
	0.032	20	40	100	100	0	25
	0.0064	0	0	55	55	0	5
	0.00128	0	0	50	10	0	0
An. gambiae [RSPH]	200	n.t.	n.t.	n.t.	n.t.	n.t.	n.t.
	100	100	100	100	100	100	100
	20	100	100	100	100	100	100
	4	100	100	100	100	100	100
	0.8	65	65	100	100	5	100
	0.16	17.5	25	100	100	0	30
	0.032	10	20	100	90	0	0
	0.0064	5	10	60	22.5	0	0
	0.00128	2.5	0	20	5	0	0
An. funestus [FUMOZ-R]	200	n.t.	n.t.	n.t.	n.t.	n.t.	n.t.
	100	100	100	100	100	100	100
	20	100	100	100	100	100	100
	4	100	100	100	100	100	100
	0.8	45	100	100	100	35	100
	0.16	0	35	100	100	10	95
	0.032	0	5	100	100	5	20
	0.0064	0	0	70	90	0	0
	0.00128	0	0	45	0	0	0
